# Effects of Fertigation Programs and Substrates on Growth, Fruit Quality, and Yield of Bell Pepper (*Capsicum annuum*) in Greenhouse Conditions

**DOI:** 10.3390/foods15030505

**Published:** 2026-02-01

**Authors:** Ángel R. Pimentel-Pujols, José M. García, Fernando Borrás, Juana Fernández-López

**Affiliations:** 1 Faculty of Agricultural and Veterinary Sciences, School of Agricultural Engineering, Post-Graduate Division, Universidad Autónoma de Santo Domingo, Santo Domingo 10013, Dominican Republic; apimentel14@uasd.edu; 2 Dominican Institute of Agricultural and Forestry Research (IDIAF), Santo Domingo 11500, Dominican Republic; jgarcia@idiaf.gov.do; 3 Statistics and Operative Research Department, Miguel Hernández University, 03202 Elche, Alicante, Spain; f.borras@umh.es; 4 Institute for Agri-Food and Agro-Environmental Research and Innovation, Miguel Hernández University (CIAGRO-UMH), Ctra. Beniel km 3.2, 03312 Orihuela, Alicante, Spain

**Keywords:** fertigation, nutrient solution, substrates, greenhouse, *Capsicum annuum*, yield, fruit quality

## Abstract

Global vegetable production exceeded 1.2 billion tons in 2022, with bell pepper (*Capsicum annuum*) accounting for 37 million tons, a crop of high value due to its versatility, commercial demand, and nutritional properties. In the Dominican Republic, greenhouse vegetable production has experienced accelerated growth over the last 23 years, reaching over 10 million m^2^ of infrastructure and increasing pepper production from 9122 to 32,000 tons. However, limitations in technical information regarding nutritional management and substrate use persist, despite the extensive empirical experience of producers and technicians. This study evaluated the effect of three fertigation programs (low, medium, and high doses: FP1, FP2, and FP3) and three substrates (carbonized rice husk- CRH, coconut fiber-CF, and a 1:1 Mix) on 180 plants grown for 141 days in a greenhouse, using a completely randomized split-plot design. Growth, physiological, quality, and yield indicators were measured. Principal Component Analysis (PCA) explained 88% of the variability, showing that FP2 and FP3, combined with BRH and the 1:1 Mix, generated greater plant height, stem diameter, chlorophyll content, and canopy development, while FP1 and CF were associated with lower performance. Regarding fruit quality, the BRH and 1:1 Mix substrates yielded higher values for length, width, and weight, whereas °Brix content responded primarily to fertigation doses. Total yield confirmed this pattern, highlighting FP3–BRH as the best combination evaluated and FP1–CF as the one with the lowest productivity.

## 1. Introduction

Global vegetable production reached approximately 1.2 billion tons in 2022, of which 37 million tons corresponded to bell pepper (*Capsicum annuum*), positioning it among the crops of greatest economic and nutritional relevance worldwide [1]. Its high demand is sustained by its culinary versatility, nutritional value, and the presence of bioactive compounds with significant nutraceutical properties [2,3,4,5,6,7,8,9]. Leading production countries, such as China, Mexico, Indonesia, Turkey, and Spain, have consolidated technified production systems that have evolved rapidly in recent decades.

In the Dominican Republic (DR), the growth of greenhouse horticulture has been particularly notable over the last 25 years. The constructed greenhouse area currently exceeds 10 million m^2^, with a sustained increase in production for both the local and export markets. Greenhouse bell pepper production has shown remarkable expansion, rising from approximately 9122 tons in 2004 to over 32,000 tons in 2023 [10]. This dynamism has positioned bell pepper as one of the main vegetables in terms of volume, commercial value, and rural employment generation. However, this intensive system faces critical challenges.

While advances have been made in plant health and general crop management, significant limitations persist in key areas such as nutrition, fertigation, and substrate use. Historically, nutritional management in the country has relied on programs adapted from international recommendations (initially from Spain), which have been empirically modified by technicians and producers due to the absence of local research. Continuous changes in genotypes, production cycles, and environmental conditions introduce variations in nutritional demand, increasing the risk of imbalances (excesses or deficiencies) that affect productivity and fruit quality, while elevating operating costs due to nutrient use inefficiency. This situation is aggravated by the limited availability of documented information regarding fertigation management and the specific substrates used in Dominican greenhouse systems. Despite the extensive accumulated experience, the lack of systematic studies hinders the formulation of evidence-based strategies to optimize agronomic and economic performance.

The province of San José de Ocoa, one of the most relevant production hubs for bell pepper, constitutes an ideal scenario to generate scientific information applicable to other production systems in the country. In this context, it is imperative to develop studies that rigorously evaluate fertigation programs and the types of substrates employed to identify their limitations and propose improvements. Nutrient use efficiency is especially critical in protected systems, where the interaction between substrate, nutrient solution, and environment determines physiological and productive behavior [5,6,7]. Optimizing nutrient use by preventing overfertilization and mitigating leaching losses is a key strategy for enhancing agricultural profitability and sustainability. Consequently, several studies have evaluated organic-based substances to minimize reliance on conventional fertilizers. In this sense, Almeyadi et al. [8] demonstrated in lettuce cultivation that liquid organic nutrient solutions enable sustainable production, reducing both operational costs and environmental impact. Furthermore, the authors confirmed that these organic treatments outperform conventional fertilization in the synthesis of secondary metabolites.

The present study addresses these gaps by evaluating the effect of different nutrition programs and substrate types on the yield and fruit quality of bell pepper produced under greenhouse conditions in San José de Ocoa. Furthermore, it seeks to generate information that contributes to the design of nutritional management plans that are better adjusted to local conditions, sustainable, and profitable, thereby strengthening the competitiveness of this value chain in the DR. The central hypothesis of this study is that the physical and hydro-physical properties of the substrate interact synergistically with fertigation programs to modulate nutrient supply efficiency and water availability.

## 2. Materials and Methods

### 2.1. Study Design

**Figure 1:**
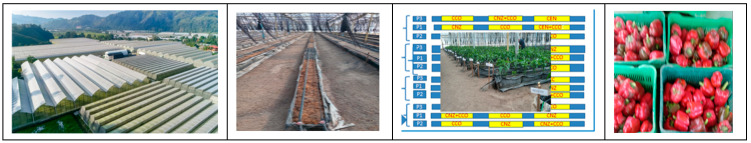
Illustrations of the study area and experimental setup.

The study was conducted in greenhouses located in San José de Ocoa (18°34′35.9″ N 70°30′18.5″ W), DR, with the objective of evaluating the effect of fertigation programs and substrate types on the growth, physiological development, quality, and fruit yield of bell pepper (*Capsicum annuum*), California type, hybrid DOTAN F1 (Enza Zaden, Almería, Spain). Prior to the experimental setup, a diagnostic analysis of nutritional management in the area was carried out through interviews, expert consultations, and a state-of-the-art review to identify current practices and define the fertigation programs to be compared. The experiment was transplanted on March 5 and lasted for 141 days. Figure 1 shows images from the study area and experimental setup.

#### 2.1.1. Evaluated Factors and Experimental Design

A 3 × 3 factorial experiment was conducted using a completely randomized split-plot design with four replications.

- Factor 1: Fertigation Programs (FP, whole plots). Three nutritional programs (FP1, FP2, and FP3) were evaluated, each divided into three phenological stages (weeks 1–4, 5–8, and 9 onwards). These programs featured progressive increments of N, P, K, Ca, Mg, S (mg/L), and micronutrients (doses of a commercial fertilizer mix), with electrical conductivity (EC) ranges between 1.2 and 2.2 dS/m (Table 1). FP2 represents the prevalent regional standard, and the upper and lower dosage limits (FP2 and FP1, respectively) were derived from this baseline, adhering to literature response ranges.
- Factor 2: Substrates (subplots). CF: 100% coconut fiber; BRH: 100% carbonized rice husk; 1:1 Mix: mixture of 50% CF + 50% BRH.

#### 2.1.2. Experimental Units

**Figure 2:**
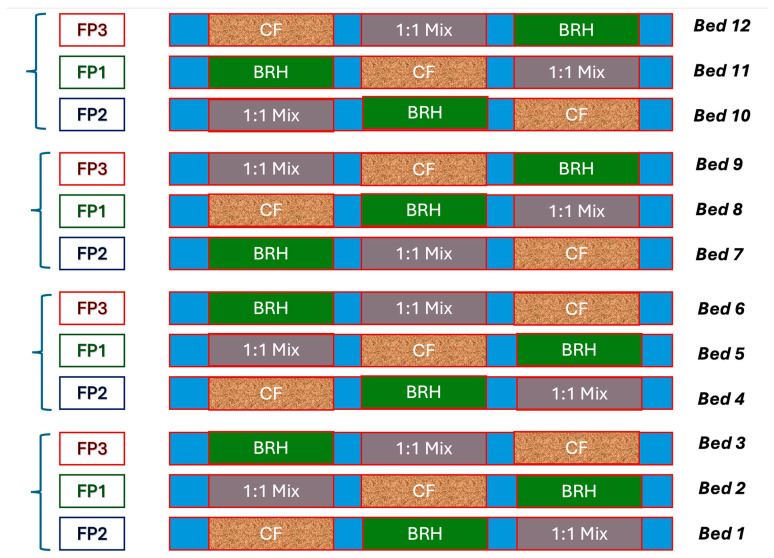
Experimental design layout. Fertigation Programs FP1-FP3 (see Table 1); CF: 100% coconut fiber; BRH: 100% carbonized rice husk; 1:1 Mix: mixture of 50% CF + 50% BRH.

The experiment consisted of 12 cultivation beds, each 9 m long, subdivided into three 3 m subplots containing the different substrates. Each main plot (corresponding to the fertigation factor) comprised 45 plants, with 15 plants assigned to each subplot (Figure 2). Five sample plants were evaluated per subplot, totaling 180 measured plants. The experimental cycle lasted 141 days after transplanting (DAT), involving eight growth measurements and seven harvests. Irrigation was applied three to four times daily, adjusted according to each phenological stage.

### 2.2. Evaluated Variables and Methodology

#### 2.2.1. Physical Characterization of Substrates

A laboratory assay was conducted to simulate the permeability and moisture retention capacity of the three selected substrates (CF: 100% coconut fiber; BRH: 100% carbonized rice husk; 1:1 Mix: mixture of 50% CF + 50% BRH). Syringes (125 mL) packed with substrate were used as test columns, upon which a drip irrigation system was simulated using burettes. The infiltration time through the vertical section, drainage duration, and water volume required to reach the saturation point (onset of drainage), as well as the final drained volume, were measured. Physical properties of the substrate were determined as follows. Bulk density was measured using the cylinder method, where 100 mL of substrate in a Pyrex^®^ cylinder was oven-dried at 105 °C for 24 h. The dry weight was recorded using a precision balance (Ohaus, Parsippany, NJ, USA; 300 g capacity), and bulk density was calculated as the ratio of dry weight to cylinder volume. Moisture content was determined by weighing 100 g of fresh substrate and drying it at 105 °C for 24 h; the moisture percentage was calculated on a wet basis using the weight difference [11,12]. Substrate pH and electrical conductivity (EC) were measured in a 1:10 (*w*/*v*) water extract using a pH/mV meter (Model MP511, Apera Instruments, Columbus, OH, USA) and an EC700 conductivity meter (Apera Instruments, Columbus, OH, USA). Nutrient solutions were monitored using a waterproof pH/EC Combo meter (Hanna Instruments, Woonsocket, RI, USA).

#### 2.2.2. Growth and Developmental Indicators in Bell Pepper Plants

These indicators were evaluated over a 141-day cycle; eight measurements were taken at 15-day intervals on 180 bell pepper plants. Crop morphology parameters such as plant height (cm), internode length (cm), and stem diameter (mm) were recorded using a metric measuring tape (cm scale) and a digital Vernier caliper (Stainless Hardened, IP54 protection, cm scale). Physiological parameters, specifically relative chlorophyll content, nitrogen status, and leaf temperature (°C) (indirect estimation), were measured using a portable Plant Nutrition Analyzer (Model GYJ-C, GOYOJO Technology, Beijing, China).

#### 2.2.3. Fruit Quality Parameters

To evaluate these parameters, 118 fruits were selected from the first harvest, conducted 85 days after transplanting (DAT), representing the interaction of the studied factors. Total soluble solids (°Brix) were determined using a digital refractometer (KRUSS Scientific, Hamburg, Germany). Fruit length and equatorial circumference were measured using a tape measure (cm).

#### 2.2.4. Yield

After harvesting, the yield or total production of each experimental unit was recorded by means of fruit counts and total weight (kg).

### 2.3. Statistical Analysis

Data were subjected to an Analysis of Variance (ANOVA) for a split-plot design, generating independent error terms for main plots, subplots, and their interaction. When the FP × S interaction was significant, simple effects were analyzed by examining the levels of one factor within the levels of the other. Otherwise, the main effects of the factors were interpreted. Mean comparisons were performed using Fisher’s Least Significant Difference (LSD) test at *p* < 0.05. Additionally, multivariate techniques were employed, including Principal Component Analysis (PCA) and Multivariate Analysis of Variance (MANOVA). When significant differences were detected, the Hotelling’s test for mean separation was applied. The database (>10,000 observations) was compiled in Microsoft Excel and analyzed using InfoStat software, 2020 version [13].

## 3. Results

### 3.1. Physical Characterization of Substrates

Results obtained from the laboratory assay of substrates (Table 2) indicated that CF reached the drainage point in a timespan 1.8 times shorter than that of BRH. Regarding retention, CF required 1.8 times less water to reach saturation compared to the 1:1 Mix and 2.4 times less than BRH. Finally, the volume drained by CF was three times greater than that recorded for BRH and the 1:1 Mix.

### 3.2. Growth and Indicators of Bell Pepper Plant Development

**Figure 3:**
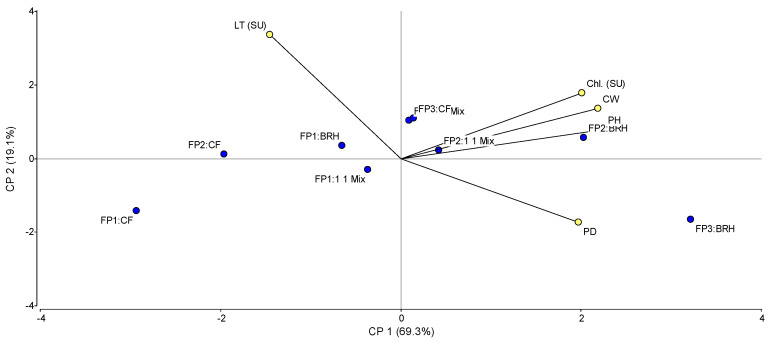
Principal Component Analysis (PCA) biplot illustrating the effect of fertigation programs (FP) and substrates on the growth variables of bell pepper (*Capsicum annuum*). Abbreviations: PH, Plant Height; SD, Stem Diameter; CW, Canopy Width; Chl, Chlorophyll content; N, Nitrogen status; LM, Leaf Moisture; LT, Leaf Temperature.

Principal Component Analysis (PCA) was performed to examine the relationship between bell pepper growth variables and the interaction of experimental factors (Figure 3). The first two principal components (PC1 and PC2) accounted for 88% of the total variability. PC1, which captured 69.3% of the variance, was defined by high positive loadings for vigor-related traits—plant height (PH, 0.50), canopy width (CW, 0.50), chlorophyll content (Chl, 0.47), and stem diameter (PD, 0.40)—and a negative loading for LT (−0.35), indicating an inverse correlation with vegetative vigor. PC2 (19.1%) was primarily dominated by a positive loading for LT (0.65) and a negative association with PD (−0.54). Regarding treatment distribution, fertigation programs FP2 and FP3 (medium and high doses) combined with the BRH substrate clustered on the positive side of PC1, indicating superior plant architecture and chlorophyll content. Conversely, the Control (CF) and FP1 treatments exhibited an opposing trend, being more closely associated with higher values of LT.

Multivariate Analysis of Variance (MANOVA) using Pillai’s trace (Table 3) indicated a significant main effect for the substrate factor (*p* = 0.0485) on the combined growth variables: plant height (PH), stem diameter (PD), canopy width (CW), chlorophyll (Chl), nitrogen (N), and leaf temperature (LT). The fertigation program (FP) showed no significant multivariate effect (*p* = 0.4281). The interaction (FP-Substrate) exhibited a marginal trend (*p* = 0.0676); therefore, univariate analyses were examined to detail specific responses.

The univariate ANOVAs revealed distinct behaviors for each variable (Table 4). Plant height was significantly influenced by the substrate and showed a significant interaction effect. Conversely, CW and Chl levels showed no significant differences for any factor. PD was strongly influenced by the substrate main effect. Similarly, leaf nitrogen was significantly affected only by the substrate. Finally, LT was significantly influenced by both the main effect of the substrate, and the interaction between fertigation and substrate.

Table 5 shows the mean comparisons for the factors that showed significant differences. For plant height and canopy width, the BRH and the mixture substrates significantly outperformed CF. Specifically, CW was also influenced by the fertigation program, where medium (FP2) and high (FP3) doses resulted in greater widths compared to the low dose (FP1). Regarding leaf nitrogen content, a clear hierarchy was observed: BRH > Mixture > CF. Conversely, leaf temperature showed an inverse trend, with the highest temperatures recorded in the CF substrate, indicative of greater water stress. In terms of interaction trends, the combination of medium or high fertigation doses with BRH consistently yielded the best vegetative performance (FP2/FP3 + BRH), while the highest leaf temperatures were associated with CF under high fertilization (FP3 + CF).

### 3.3. Fruit Quality Parameters

**Figure 4:**
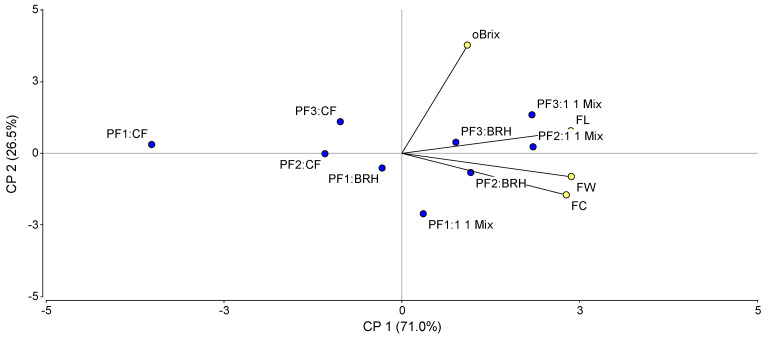
Principal Component Analysis (PCA) of the effect of fertigation programs (FP1, FP2 and FP3) and substrates (coconut fiber, CF; carbonized rice husk, BRH; and 1:1 Mix) on the quality of bell pepper fruit (length, FL; Circumference, FC; weight, FW; and °Brix content, °Brix).

The Principal Component Analysis (Figure 4) indicated that combinations of medium and high fertigation programs with carbonized rice husk and the mixture (specifically FP3–BRH, FP3–1:1 Mix, and FP2–BRH) were associated with the highest values for °Brix, fruit width, length, and weight. Conversely, combinations linked to the low fertigation level with coconut fiber (notably FP1–CF) exhibited an opposite trend, showing the lowest performance regarding the magnitude of these variables.

The Analysis of Variance (ANOVA) revealed significant differences among substrates regarding fruit length, width, and weight. No significant differences were observed for total soluble solids content (°Brix). The results of the mean comparison of the evaluated variables in bell pepper (weight, length, width, and °Brix) according to the significance of the corresponding factor are shown in Table 6.

The substrate type significantly influenced (*p* < 0.0001) the physical characteristics of the fruit. Fruits grown in the 1:1 Mix exhibited the highest average weight (227.49 g), statistically outperforming the BRH treatment (210.00 g) and CF, which yielded the lowest weight (172.53 g). Regarding fruit dimensions, both the 1:1 Mix and BRH substrates showed similar performance, producing significantly longer and wider fruits compared to those grown in CF. Conversely, total soluble solids were not affected by the substrate type (*p* = 0.9545); however, a significant response to the fertigation program was observed (*p* = 0.0761). The high fertigation level (FP3) resulted in fruits with higher sugar content (6.65 °Brix) compared to the low level (FP1, 6.36 °Brix), with no significant differences observed relative to the medium level (FP2).

### 3.4. Fruit Yield Analysis by Harvest

The first harvest was conducted 85 days after transplanting (DAT). A total of seven harvests were performed between 85 and 141 DAT. These were carried out at approximately 7-day intervals, except for the final harvest, which was conducted after 14 days. Production dynamics showed variability influenced by the evaluated factors over time. Table 7 shows a summary of the ANOVA (*p*-values) and means comparison trends for bell pepper yield across seven harvest events. For the first harvest (85 DAT), no significant effects for the interaction or the main effects of fertigation programs and substrates were observed. For the second harvest (92 DAT), no interaction or fertigation effects were observed. However, a significant effect was found for the substrates. The 1:1 Mix and CF outperformed BRH, with no significant differences between them. For the third harvest (99 DAT), significant effects were observed for the FP × S interaction, with no differences in the main effects. The combination of the high-level fertigation program (FP3) with BRH significantly outperformed the other combinations. For the fourth harvest (105 DAT), significant differences were recorded only for the substrate factor. BRH and the 1:1 Mix outperformed CF, showing no statistically significant differences between each other. For the fifth harvest (113 DAT), a similar trend to the previous harvest was observed. BRH significantly outperformed both the 1:1 Mix and CF, with no significant differences found between the latter two. For the sixth harvest (127 DAT), significant differences were found for both the interaction and the main effect of the substrate. In the interaction means separation, the medium and high fertigation programs (FP2 and FP3) combined with BRH and the 1:1 Mix resulted in the highest yields (kg m^−2^). For the seventh harvest (141 DAT), in this final evaluation, only the substrate factor showed statistical significance: BRH and the 1:1 Mix outperformed CF. It should be noted that while the evaluation period concluded, the crop continued to produce.

**Figure 5:**
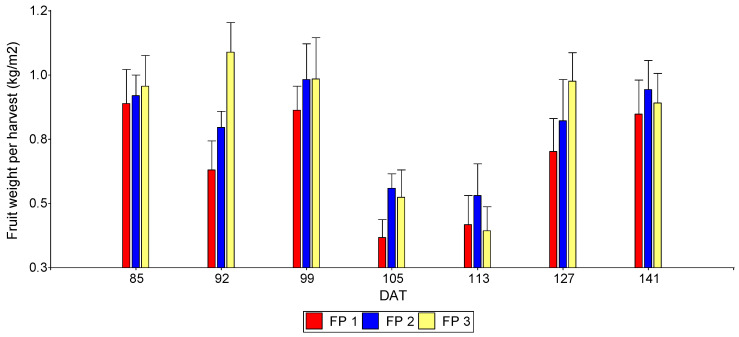
Bell pepper fruit yield across seven harvests according to fertigation program under greenhouse conditions. DAT: days after transplanting; FP1: Fertigation Program 1; FP2: Fertigation Program 2: FP3: Fertigation Program 3 (see Table 1).

As general trends in this evaluation, the following could be said: the lowest productivity was visualized in harvests 4 and 5 (105 and 113 DAT) (Figure 5). Furthermore, the low-dose fertigation program (FP1) consistently showed inferior performance at 92, 105, and 127 DAT (Figure 5). Regarding the total accumulated yield of the seven harvests, marginally significant effects were recorded for the interaction and the fertigation program, while highly significant effects were observed for the substrate factor (Table 7).

### 3.5. Total Fruit Yield

The yield obtained from each of the seven harvests was accumulated for each experimental unit to calculate the total fruit yield, expressed in kg m^−2^. An Analysis of Variance (ANOVA) was performed on the logarithmically transformed data to meet the assumptions of normality and variance homogeneity. The results are shown in Table 8.

**Figure 6:**
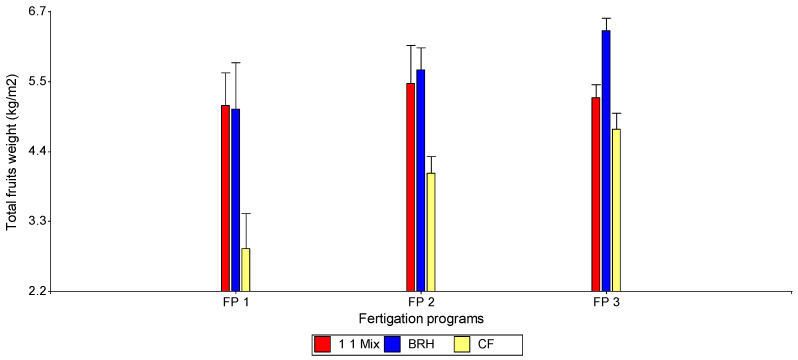
Total bell pepper fruit yield according to the interaction between substrate type (CF, BRH, and 1:1 Mix) and fertigation program (FP1, FP2, and FP3).

Regarding total yield (Table 8), the combination of the high-level fertigation program and carbonized rice husk (FP3–BRH) significantly outperformed all other treatments. Conversely, the lowest yield was recorded for the combination of the low-dose program and coconut fiber (FP1–CF). The 50/50 substrate mixture (1:1 Mix) exhibited an intermediate effect across the three evaluated fertigation programs. It was observed that the performance of CF was consistently inferior to that of the 1:1 Mix and BRH across all fertigation levels (FP1, FP2, and FP3). Notably, BRH outperformed both CF and the 1:1 Mix specifically under the highest fertigation dose (Figure 6).

## 4. Discussion

### 4.1. Growth Plant Indicators

Regarding the performance of the studied factors, the program with the highest and medium nutrient doses (FP3 and FP2), combined with carbonized rice husk (BRH) and, secondarily, with the substrate mixture (1:1 Mix), exhibited the best performance in growth variables such as stem diameter, plant height, canopy width, and foliar levels of nitrogen and chlorophyll (SPAD). These results coincide with studies reporting superior chlorophyll content in pepper plants treated with medium and high nitrogen levels in fertigation [14]. This consistency suggests that the higher nitrogen availability in the rhizosphere directly stimulated the biosynthesis of chlorophyll and structural proteins, enhancing the photosynthetic capacity required for vegetative expansion. Furthermore, it is plausible to hypothesize that the physical properties of BRH facilitated greater ion retention compared to other substrates, maximizing the nutrient interception efficiency suggested by Angulo-Guillén [14].

Conversely, the poorest performance was recorded with the combination of low and medium doses applied to coconut fiber (CF), which is related to reductions in growth indicators when nutrient concentration in the solution is decreased [15]. However, in our study, this negative effect appears to have been exacerbated by the high drainage rate of the CF. Unlike the conditions in Wang’s study, where nutrient reduction was a management strategy, in our trial, the combination of a “low dose” with a low-retention substrate (CF) likely caused accelerated leaching, driving the nutrient concentration in the root zone below the critical threshold necessary for cell division and biomass maintenance.

These differences are directly related to the physical properties of the substrates (Table 3), which condition the retention, availability, and movement of water and nutrients. CF showed a more heterogeneous particle distribution, favoring greater drainage and, consequently, lower moisture and nutrient retention. The literature supports that coconut fiber, due to its porous structure and composition, can be outperformed by substrates such as rockwool in growth and developmental variables, including chlorophyll content (SPAD) [16]. Furthermore, other comparative studies showed that rice husk resulted in superior dry weight for pepper leaves and stems, although coconut fiber excelled in stem diameter [17]. Our results, where BRH outperformed CF in all biometric variables, contrast with that finding. We hypothesize that this difference lies in the material processing: unlike the standard husk, the BRH used in this study possesses a thermally generated microporous network. This structure enhances water retention and overcomes the particle-size-dependent hydration limitations described by Ruiz-Espinoza et al. [18], allowing for a more stable water supply than CF, which exhibited excessive drainage under our conditions.

In contrast, BRH and the 1:1 Mix, possessing a more homogeneous structure, retained greater moisture and nutrients for a longer period, thus favoring vegetative growth. The hydration behavior of coconut fiber depends closely on the size, proportion, and distribution of its constituent particles [18]. The highest nutrient dose resulted in the best growth performance, followed by the medium dose. This result is expected, as higher nutrient concentrations, up to a certain optimal threshold (e.g., 2 dS m^−1^ electrical conductivity in pepper), stimulate greater growth [19,20]. However, it has been reported that excessively high irrigation and nitrogen doses can negatively affect quality [21].

Although Wang et al. [20] warn that high nitrogen and irrigation doses can reduce nutrient use efficiency and fruit quality, in our trial, the high dose was not detrimental. one could suggest that given the high macroporosity and drainage rate of these substrates, the FP3 regime functioned as a hydro-nutritional compensation mechanism. The higher input concentration counteracted the leaching effect, ensuring that the actual ion availability in the rhizosphere remained at levels sufficient to sustain crop demand without reaching toxicity levels.

SPAD measurements recorded in this study fell within an optimal range of 50 to 60 units, validating the nutritional status of the crop. The rice husk charcoal substrate and the high-dose fertigation program yielded the highest chlorophyll readings, demonstrating a positive plant response to increased nutrient availability. These results align with the close correlation between chlorophyll and nitrogen reported by Cruz-Durán et al. [22], who establish SPAD readings as a reliable indicator for diagnosing nitrogen in plant tissue. However, that author reported optimal responses with medium nitrogen doses, which contrasts slightly with the superiority of the high dose observed in our research, possibly due to differences in nutrient retention among the substrates used. This hypothesis is supported by Ruiz-Espinoza et al. [20], who note that substrate properties condition the growth. Therefore, contrary to the warnings by Wang et al. [21] regarding the inefficiency of high doses, our system likely required this higher input to compensate for leaching and maintain adequate foliar levels. On the other hand, a study conducted on basil (*Ocimum basilicum*) using increasing nitrogen doses demonstrated a close relationship between SPAD readings, chlorophyll content, and plant nitrogen concentration. These authors reported determination coefficients close to one [23], validating the use of these non-destructive measurements to estimate the nutritional status of the crop.

Furthermore, Soto-Bravo and Angulo-Guillén [24] confirm the utility of SPAD transmittance as a non-destructive estimation method. However, they note that the accuracy of these indices for estimating actual chlorophyll content is higher when nitrogen levels are low (stress conditions), with reduced sensitivity at sufficiency levels.

This suggests that while our values of 50–60 confirm the absence of deficiencies, the linearity between SPAD values and nitrogen content may plateau at these high fertilization ranges, potentially masking the detection of “luxury consumption” beyond the optimal threshold. In this study, stem diameter exceeded 10 mm across both substrate types and irrigation programs. Independently, the rice husk charcoal substrate and the high fertigation dose yielded slightly higher averages; a similar trend was recorded for plant height. Regarding stem diameter, these findings align with those reported by Tuckeldoe et al. [25] for pepper cultivars grown in coconut coir substrate, although their results for plant height were lower than those achieved in the present study. These authors maintain that stem development is related to nutritional status, emphasizing that high phosphorus and calcium levels may have influenced the observed differences. Therefore, it could be hypothesized that the greater height obtained in our trial suggests that the FP3 provided superior bioavailability of these macro-elements compared to the reference study. This supports the high-dose fertigation program employed in this study, which represents the highest nutrient input.

### 4.2. Fruit Quality Indicators

For the variables regarding fruit width, weight, length, and total soluble solids content (°Brix), the best indicators were obtained with the medium and high doses combined with the 1:1 Mix, as well as with the medium dose applied to BRH. The lowest values were observed with the low dose combined with CF. Once again, a strong influence of the CF substrate is evident, as its physical characteristics (high heterogeneity and coarse porosity) favor leaching and reduce fertigation uptake efficiency.

Regarding fruit morphometry, lengths ranged from 130 to 150 mm. The rice husk charcoal substrate yielded the highest values, whereas coconut fiber resulted in the lowest. These findings surpass the quality standards reported by Monge-Pérez and Loría-Coto [26], who classify fruits exceeding 120 mm in length as first-class quality, thus highlighting the commercial viability of the substrates evaluated in this study. This validates the physical superiority of the BRH substrate; unlike the density restrictions noted by Monge-Pérez and Loría-Coto [26] that limit fruit expansion, this substrate provided optimal root volume, ensuring that fruit elongation was not hindered by physical constraints.

Several factors influence Brix levels, most notably nutrition, moisture content, electrical conductivity, and plant stress [27]. While the literature reports normal average levels of around 6 °Brix, the values obtained in this study exceeded this benchmark. This increase was attributed to distinct mechanisms depending on the factor evaluated: regarding the high fertigation dose, the increase resulted from greater nutrient availability and accumulation of soluble solids; whereas for the coconut coir substrate, the elevated values were likely related to water stress experienced by the plants. This distinction is crucial for interpreting quality: while Grupoproin [27] identified the nutrition as a primary driver, our results clarify that high °Brix in coconut fiber is partially a symptom of dehydration (concentration by volume reduction), whereas in the high-dose treatment, it represents genuine solute accumulation.

Despite the low total yield, the CF substrate presented the highest values for °Brix, a phenomenon likely associated with lower water content in fruits produced under conditions of greater water stress and rapid drainage. This finding is in agreement with studies that also report higher °Brix levels in coconut fiber compared to rice husk mixtures or rockwool [18]. Regarding the fertigation program, the high dose produced the best °Brix values, aligning with research showing a positive response to nutrients in improving pepper quality indicators [28,29]. Specifically, Liang et al. [28] emphasize the correlation between N-P-K availability and fruit sweetness in soil; our findings extend this to soilless culture, confirming the observation of Rahman et al. [29] that in hydroponic systems, maintaining high nutrient concentrations in the solution is the direct driver for maximizing metabolic quality attributes like TSS.

### 4.3. Harvest and Total Fruit Yield

In the analysis of the seven harvests, a clear dominance of the substrate factor was observed from the second harvest onwards, with BRH and the 1:1 Mix consistently out-performing CF in yield. This instability of CF in controlling moisture content, and consequently yield, has been reported by other authors [30]. Specifically, Yeo et al. [30] highlight that while coir is a standard medium, its hydraulic properties require precise nutrient management; the lower yields obtained in CF suggest that its rapid drainage led to nutrient leaching that could not be compensated, affecting total weight. Other studies have shown that substrate mixtures, such as a combination of 50% rice husk and 50% coconut fiber, can yield better productive results [31]. This aligns with the results obtained for the 1:1 Mix, confirming Guerrero’s [31] hypothesis that combining substrates creates a synergistic effect, balancing the low retention of coarse fibers with the stability of denser materials. In this regard, Almeida Garcia et al. [32] report that bell pepper harvest begins between 90 and 100 days after transplanting, with a weekly harvest frequency and average yields ranging from 11 to 19 kg·m^−2^. These productive and phenological parameters closely match the behavior observed in the present study, validating the results obtained under our experimental conditions. By falling within the range described by Almeida Garcia et al. [32], our study demonstrates that despite the substrate variations, the overall agronomic management was adequate, allowing the specific effects of the treatments to be isolated without phenological anomalies. This structural dependence is reinforced by Mejía-Pérez et al. [33], who identify the substrate–nutrient interaction as a critical determinant starting from the seedling stage. Our findings extend this conclusion to the full productive cycle, confirming that the physical stability provided by the BRH substrate is essential for translating early potential into final harvest yield, avoiding the losses associated with the excessive drainage observed in CF.

Regarding bell pepper yield, the maximum value achieved with the highest fertigation dose and carbonized rice husk (FP3-HBR) reached 6.9 kg m^−2^. Although this figure is lower than the 10 kg m^−2^ reported in reference studies such as Mejía-Pérez et al. [33], it is crucial to note that the present experiment was terminated at the seventh harvest, while the crop remained physiologically active and productive. In contrast, cited reference values typically correspond to completed crop cycles. Consequently, the partial accumulated yield suggests that the FP3-HBR treatment demonstrates a competitive production potential comparable to reported averages, had the crop cycle been extended.

Regarding total yield (sum of the seven harvests), a marked effect of the substrate was evidenced, followed by the fertigation program and the interaction between both. The superiority of substrate mixtures (rice husk with rockwool) over coconut fiber in pepper production has been previously reported [34]. This agrees with the work of Inden and Torres [34], who attributed the success of mixtures to the balance between water retention and aeration, a physical equilibrium that our BRH substrate achieved better than the rapidly draining CF. The trend of BRH and the highest fertigation dose remained consistent. Increasing the nutrient dose above the standard, up to a limit, has been demonstrated to increase the proportion and yield of pepper fruits [30,35,36]. Additionally, it has been found that a concentration of 100% of the standard nutrient solution (based on Hoagland’s solution) can contribute to the best results in pepper [37]. This consensus across studies [30,35,36] validates this high-dose strategy, confirming that during peak fruiting, the crop’s “sink strength” requires nutrient concentrations exceeding standard recommendations to prevent abortion and maximize fruit load, as observed with the 100% solution efficacy noted by De Oliveira et al. [37].

A study conducted by Fortis-Hernández et al. [38] compared different organic substrates and a sand control using the Steiner solution. In their research, the control treatment yielded the highest production and Brix levels, with the first harvest carried out 90 days after transplanting. The present study aligns with these findings regarding the phenological timing of the first harvest, fruit Brix degrees, and yield, outcomes that are consistent with the use of a superior nutrient solution derived from the Steiner formulation. This validates the robustness of the Steiner formulation used in both studies; its specific ionic balance appears to be the primary factor synchronizing phenological stages and metabolic accumulation (Brix), regardless of the substrate difference (organic vs. sand). Another study conducted by Villa-Castorena et al. [39] evaluated different millimolar nutrient concentration levels, finding that fruit length, width, and weight were positively affected by high doses. In their research, the lowest millimolar concentration solution produced the lowest fruit weights, whereas medium and high concentrations showed no significant differences between them. Similar patterns were observed in the present study regarding productivity and the response to medium and high solutions. Notably, this study achieved average fruit weights 6% higher than those reported by these authors. This superior weight performance could be attributed to the physicochemical properties of the rice husk charcoal; unlike the inert media used by Villa-Castorena et al. [39], the porous structure of BRH may have facilitated better rhizosphere oxygenation and nutrient uptake efficiency, translating into heavier individual fruits.

Finally, the evaluation of substrate permeability at the laboratory level (Table 2) showed that CF drained faster, required less water to reach saturation, and had a higher drained volume compared to the 1:1 Mix and BRH. This corroborates the finding that the predominance of macropores in CF generates a faster water flow and lower water retention, in contrast to the more balanced structure of BRH and the Mix [40]. Other studies maintain that coconut fiber, although presenting lower bulk density and higher porosity, has a lower moisture retention capacity, and its improvement requires optimizing the size and distribution of its particles [41,42]. Therefore, its use is recommended in mixtures and with better homogenization, as its individual use can result in low yields [43].

The decrease in growth and yield in the CF substrate can be explained by its fast drainage, which impose intermittent drought stress. This condition is critical, according to Alvino et al. [44], who note that pepper plants are particularly sensitive due to their pronounced leaf area and high stomatal conductance, factors that amplify water loss through transpiration. Under these water deficit conditions, the primary physiological mechanism affected is photosynthesis. Water deficiency directly hinders carbon assimilation by limiting CO_2_ diffusion to carboxylation sites due to a combined reduction in stomatal and mesophyll conductance. As reported by Flexas et al. [45], this diffusive limitation is considered the main environmental factor restricting photosynthesis and, consequently, biomass production.

At the cellular level, this drought stress disrupts vital processes such as cell division and expansion, leading to a substantial reduction in shoot and fruit size, as noted by Ntanasi et al. [46]. Additionally, the low moisture retention in the CF substrate impairs mineral nutrition; as the water content in the medium decreases, the diffusion of nutrients from the substrate to the root surface slows down, reducing the uptake of essential elements [47]. In addition, the suppression of photosynthetic activity and limited nutrient absorption [46,47] would justify the lower yields observed in CF compared to the substrates containing rice husk, where moisture levels remained more stable.

In synthesis, these findings confirm that a substrate structure with a better air–water balance, uniformity, and homogeneity in pore distribution, combined with adequate nutrient availability (high or medium doses), promotes the better growth and development of bell pepper, translating into higher yields and better fruit quality.

## 5. Conclusions

This study concludes that while both fertigation and substrate significantly influence the agronomic performance of the greenhouse bell pepper, the substrate type emerged as the dominant factor, conditioning the efficacy of fertilization programs due to the variability of its physical properties.

Regarding vegetative growth, the interaction between factors was significant. Medium (FP2) and high (FP3) fertigation doses combined with carbonized rice husk (BRH) or the mixture (1:1 Mix) optimized key variables such as plant height, stem diameter, and chlorophyll content. Conversely, the use of coconut fiber (CF), particularly with low doses, was correlated with reduced vigor and inferior biometric response.

With respect to fruit physical quality (weight, length, and width), the response was driven almost exclusively by the substrate. BRH and the 1:1 Mix favored greater fruit development, whereas CF consistently presented the lowest values, attributable to its high macroporosity and lower water and nutrient retention. Internal quality (°Brix) showed no statistically significant variations, indicating that fruit sweetness is less sensitive to these factors compared to physical traits.

The temporal analysis of production revealed that the influence of the substrate intensified as the crop cycle progressed. Although initial harvests were uniform, CF demonstrated consistently inferior performance during the intermediate and final stages. A positive synergy was confirmed in the total accumulated yield: the combination of the high fertigation dose (FP3) with carbonized rice husk (BRH) generated the highest productivity, significantly outperforming all other combinations.

From an economic perspective, although the FP3 program increased fertilizer costs to 2.0 USD m^−2^ (compared to 1.7 USD m^−2^ for FP2), this expense is justified by the agronomic response. The FP3-BRH interaction not only maximized yield compared to FP2-BRH (6.6 vs. 5.9 kg m^−2^) but also demonstrated superior economic efficiency, achieving a 9% higher profitability margin per m^2^. Therefore, FP3-BRH is confirmed as the most viable strategy, as the value of the additional yield effectively offsets the higher input costs.

Finally, it is concluded that the physical limitations of coconut fiber (excessive porosity and drainage) acted as a restrictive factor that could not be compensated for even by increasing the nutrient dose. Therefore, under the studied conditions, the use of carbonized rice husk (BRH) under a high-dose fertigation program (FP3) is recommended as the most suitable strategy to maximize the productive potential of bell pepper in greenhouse systems.

## Figures and Tables

**Table 1:** Fertigation programs (mg/L) evaluated for bell pepper according to phenological stages.

Fertigation Program (FP)	Phenological Stage	N	P	K	Ca	Mg	S	EC *	Weeks	Micros **
FP1	1	90	30	163	96	36	65	1.2	1–4	35
FP1	2	95	35	175	104	39	73	1.3	5–8	35
FP1	3	100	40	192	111	42	78	1.4	9----	35
FP2	1	110	35	217	125	48	85	1.6	1–4	45
FP2	2	120	40	229	132	51	95	1.7	5–8	45
FP2	3	130	45	242	143	54	100	1.8	9----	45
FP3	1	145	40	258	150	57	105	1.9	1–4	55
FP3	2	155	45	267	157	60	110	2.0	5–8	55
FP3	3	170	50	300	175	66	120	2.2	9----	55

Stage: 1 (weeks 1–4), 2 (weeks 5–8), 3 (9 onwards); EC *: Electrical conductivity (dS/m); Micros **: application rate, in grams, of a commercial micronutrient fertilizer mixture.

**Table 2:** Physical characteristics and permeability test of substrates.

Substrate	Water Consumed to Saturation (mL)	Infiltration Time (s)	Drained Water (mL)	Moisture (%)	Bulk Density (g cm^−3^)
CF	56	20	15.5	13.28	0.1236
BRH	135	36	5.5	22.07	0.2049
1:1 Mix	100	25	5.0	18.37	0.1765

CF: coconut fiber; BRH: carbonized rice husk; 1:1 Mix: mixture of 50% CF + 50% BRH.

**Table 3:** Multivariate Analysis of Variance (MANOVA) results using Pillai’s Trace for the evaluated growth variables.

	Statistic	F Value	Num df	Den df	*p*
FP	0.96	1.11	10	12	0.4281
Substrate	0.84	2.18	10	30	0.0485
FP × Substrate	1.30	1.64	20	68	0.0676

FP: Fertigation Program.

**Table 4:** Summary of the Analysis of Variance (ANOVA) for the effects of fertigation, substrate, and their interaction on bell pepper growth variables.

	FP	Substrate	FP × Substrate
PH	0.8465 ns	0.0002 **	0.0583 *
PD	0.8674 ns	0.6563 ns	0.2314 ns
CW	0.5118 ns	0.0004 **	0.3430 ns
Chfl	0.5122 ns	0.1744 ns	0.4077 ns
N	0.4683 ns	0.0274 *	0.3158 ns
LT	0.8881 ns	0.0354 *	0.0237 *

PH: plant height; PD: steam diameter; CW: canopy width; Chfl: chlorophyll; N: leaf nitrogen; LT: leaf temperature. FP: fertigation programs. ns: not significant; * significant at *p* < 0.05; ** highly significant at *p* < 0.01.

**Table 5:** Mean comparison of bell pepper growth variables for the studied factors and interaction trends.

	FP	Substrate	FP × Substrate
PH	NS	CF < Mix 1:1 = BRH	FP3 BRH
CW	FP1 < FP2 = FP3	CF < Mix 1:1 = BRH	FP3 FP2/BRH
N	NS	CF < Mix 1 1 < BRH	FP2 FP3/BRH
LT	NS	BRH = Mix 11 < CF	FP3 CF

PH: plant height; CW: canopy width; N: leaf nitrogen; LT: leaf temperature. FP1, FP2, FP3: 1, 2 and 3 fertigation programs; BRH: carbonized rice husk; CF: coconut fiber; Mix 1:1: mixture of BRH and CF (50:50 *v*/*v*). Statistic: “=” indicates no statistical difference between means; “<” indicates a significant difference (*p* < 0.05). The “FP **×** Substrate” column highlights the treatment combinations.

**Table 6:** Effect of substrate type on the physical characteristics of bell pepper fruits (*Capsicum annuum* L.).

Substrate	Fruit Weight (g)	Fruit Length (cm)	Circumference (cm)
CF	172.53 ± 5.45 c	13.65 ± 0.22 b	25.77 ± 0.30 b
BRH	210.00 ± 5.76 b	14.66 ± 0.23 a	27.38 ± 0.32 a
1:1 Mix	227.49 ± 6.11 a	15.05 ± 0.25 a	27.70 ± 0.33 a
*p*-value	0.0001	0.0001	0.0001

Data are presented as mean ± standard error. CF: coconut fiber; BRH: carbonized rice husk; 1:1 Mix: 50% CF + 50% BRH mixture. (a–c) Different letters within the same column indicate statistically significant differences according to Fisher’s LSD test (*p* < 0.05).

**Table 7:** Summary of Analysis of Variance (*p*-values) and means comparison trends for bell pepper yields across seven harvest events.

Harvest	DAT	*FP × S	*FP	*S	Main Findings
1	85	0.5380	0.7251	0.3075	No significant differences observed.
2	92	0.2254	0.1149	0.0061 *	1:1 Mix = CF > BRH
3	99	0.0515 *	0.8004	0.3040	FP3 + BRH outperformed others.
4	105	0.8999	0.2564	0.0012 *	BRH = 1:1 Mix > CF
5	113	0.3574	0.6325	0.0024 *	BRH > 1:1 Mix = CF
6	127	0.0737	0.1678	0.0125 *	(FP2/FP3) + (BRH/Mix) best yield.
7	141	0.3128	0.8718	0.0001 *	BRH = 1:1 Mix > CF
Total	--	0.1084	0.0972	0.0001 *	1:1 Mix = BRH > CF

DAT: Days after transplant. *p*-values with * indicate statistical significance (*p* < 0.05 or marginal *p* < 0.10 where noted). Symbols (>, =) describe the statistical hierarchy between treatments according to the LSD test. *FP × S: Effects of the interaction between fertigation programs and substrates; *FP: main effect of fertigation programs; and *S: main effect of substrates; CF: coconut fiber; BRH: carbonized rice husk; 1:1 Mix: 50% CF + 50% BRH.

**Table 8:** Mean comparison of the interaction between fertigation programs and substrates on total bell pepper fruit yield (kg m^−2^).

Fertigation Program	Substrate	Total Fruit Yield (kg m^−2^)
FP1	CF	2.75 a
FP2	CF	4.17 b
FP3	CF	4.68 bc
FP1	BRH	5.01 bcd
FP2	BRH	5.89 cd
FP3	BRH	6.61 d
FP1	1:1 Mix	5.13 bcd
FP2	1:1 Mix	5.50 cd
FP3	1:1 Mix	5.37 bcd

FP: fertigation program, CF: coconut fiber, BRH: carbonized rice husk, 1:1 Mix: 50% CF + 50% BRH. Statistical analyses and mean separation were performed on the log-transformed data; the values presented correspond to back-transformed means. Different lowercase letters indicate significant differences (*p* < 0.05) according to LSD’s test performed on log-transformed data.

## Data Availability

The original contributions presented in the study are included in the article, further inquiries can be directed to the corresponding author.
